# The Epithelial Cell Glycocalyx in Ocular Surface Infection

**DOI:** 10.3389/fimmu.2021.729260

**Published:** 2021-08-23

**Authors:** Pablo Argüeso, Ashley M. Woodward, Dina B. AbuSamra

**Affiliations:** Department of Ophthalmology, Schepens Eye Research Institute of Massachusetts Eye and Ear, Harvard Medical School, Boston, MA, United States

**Keywords:** epithelium, glycocalyx, mucins, infection, ocular surface

## Abstract

The glycocalyx is the main component of the transcellular barrier located at the interface between the ocular surface epithelia and the external environment. This barrier extends up to 500 nm from the plasma membrane and projects into the tear fluid bathing the surface of the eye. Under homeostatic conditions, defense molecules in the glycocalyx, such as transmembrane mucins, resist infection. However, many pathogenic microorganisms have evolved to exploit components of the glycocalyx in order to gain access to epithelial cells and consequently exert deleterious effects. This manuscript reviews the implications of the ocular surface epithelial glycocalyx to bacterial, viral, fungal and parasitic infection. Moreover, it presents some ongoing controversies surrounding the functional relevance of the epithelial glycocalyx to ocular infectious disease.

## Introduction

The glycocalyx is a carbohydrate-rich coating present on the external surface of plasma membranes. It serves as a barrier against pathogens but, at the same time, can be utilized by such pathogens for attachment and entry. The major components of the glycocalyx are glycans composed of monosaccharides linked to each other with various degrees of structural complexity. Glycans can be classified according to the internal organization of monosaccharides and the nature of the linkage established with protein and lipid moieties on plasma membranes. O-glycans are commonly attached to the hydroxyl group of serine or threonine residues on proteins, whereas N-glycans are linked *via* an amide linkage to asparagine. Glycosaminoglycans contain repeating disaccharide units that are either free or covalently attached to core proteins to form proteoglycans. Glycosphingolipids and glycosylphosphatidylinositols consist of a hydrophobic lipid tail attached to a glycan moiety, with the glycan moiety of glycosylphosphatidylinositols covalently linked to a variety of proteins (1).

The transparent cornea together with the associated tear film is the primary refractive surface of the visual system that allows the passage of light onto the retina for clear vision. It is surrounded and maintained by the adjacent corneoscleral limbus and the connective tissue of the conjunctiva with its adnexa. The outermost layer of the cornea and conjunctiva is composed of a non-keratinized stratified squamous epithelium and constitutes the first cellular barrier against pathogen penetrance. Apical membranes on the most apical cell layer exhibit folds or ridges, termed microplicae, containing a prominent glycocalyx rich in transmembrane mucins, proteoglycans and glycosphingolipids. Components of this glycocalyx extend hundreds of nanometers above the plasma membrane and interface with the external environment (2). Apical cells also exhibit tight junctions that regulate the paracellular movement of molecules and microorganisms across the epithelium. The surface of the eye, like other mucosal tissues in the human body, represents a route of transmission for many bacteria, viruses, fungi and parasites. Here, we provide a brief review of major components of the ocular surface epithelial glycocalyx and their involvement in resisting or facilitating infection.

## The Glycocalyx in Ocular Surface Infection

### Bacterial Infection

Bacterial keratitis is a sight-threatening infectious disease of the cornea caused by different types of bacteria, including *Staphylococcus aureus, Streptococcus pneumoniae* and *Pseudomonas aeruginosa* (3). One of the first steps during bacterial infection is the adhesion of the pathogen to glycoproteins and glycolipids on the epithelial cell glycocalyx through specific glycan recognition mechanisms (4, 5). Notably, this adhesive interaction does not take place unless the epithelia are damaged (6). Mucins stand out among the multiple protective components of the healthy glycocalyx because of their ability to limit infectious disease while accommodating resident microbiota (7). Transmembrane mucins have large and rigid extracellular domains that extend 500 nm or more above the cell surface. They can prevent microbial colonization by several mechanisms that include forming a physical barrier, acting as adhesion decoys and, in certain cases, exposing specific glycans that attenuate pathogen virulence.

The presence of the transmembrane mucin MUC16 at the ocular surface is a major restrain to the passage of bacteria. Reports using a human cell culture model of stratified corneal epithelium have evidenced that MUC16 and mucin O-glycans protect the epithelial surface against *S. aureus* adhesion (8, 9). It has been established that transmembrane mucins in cornea play a crucial role in the early response against pathogens by suppressing Toll-like receptor signaling and the expression of the proinflammatory cytokines (10). Therefore, in the absence of pre-existing defects or wounding, non-opportunistic bacteria must rely on the enzymatic removal of transmembrane mucins to access epithelial cells and cause disease. This is the case with epidemic disease-causing *S. pneumoniae* species, which secrete a metalloproteinase, ZmpC, that selectively induces ectodomain shedding of MUC16, leading to loss of glycocalyx barrier function and enhanced bacterial internalization (11, 12). Under homeostatic conditions, the barrier function of MUC16 in the epithelial glycocalyx is reinforced by galectin-3, a multimerizing lectin that causes carbohydrate-dependent crosslinking of transmembrane mucins (13–15). Intriguingly, galectin-3 is highly expressed in the human cornea and serves as a ligand for *P. aeruginosa* lipopolysaccharide (16). A potential explanation for the scarcity of adhesive events leading to *P. aeruginosa* infection in the healthy eye is that the strong, high-affinity association between mucins and galectin-3 on the glycocalyx provides steric hindrance, therefore interfering with the ability of the bacterium to interact with the lectin.

Some bacteria exploit proteoglycans present on the ocular surface epithelia and underlying extracellular matrix to promote infection. Syndecan-1 and perlecan are two proteoglycans containing chains of heparan sulfate and chondroitin sulfate—major glycosaminoglycans found in the human cornea (17). Syndecan-1 is known to enhance the attachment of *S. aureus* to the plasma membrane of several cell types but fails to do so in corneal epithelial cells. Instead, *S. aureus* induces shedding of syndecan-1 from the glycocalyx of corneal epithelial cells to produce ectodomains that interfere with the capacity of neutrophils to kill the bacteria (18). Similarly, the ability of *P. aeruginosa* and *S. pneumoniae* to infect the cornea is not associated with direct adhesion to proteoglycans present on the epithelial glycocalyx. *P. aeruginosa* preferably binds to perlecan exposed in the basement membrane after corneal injury (19, 20), whereas *S. pneumoniae* adhesion relies on the ability of syndecan-1 to promote the assembly of fibronectin fibrils in the basement membrane (21).

The clearance of bacteria can be enhanced by glycans and glycoconjugates present in the tear fluid bathing the ocular surface epithelia. For example, it has been shown that binding of *P. aeruginosa* to N-glycans on tear glycoproteins functions as a protective mechanism that reduces bacterial adhesion and infection, most likely by facilitating the removal of the bacteria through the tear drainage system (22). Similarly, studies have shown that surfactant protein D, a collagen-containing calcium-dependent lectin with ability to bind lipopolysaccharide from Gram-negative bacteria, is present in the tear fluid and protects against *P. aeruginosa* invasion (23, 24).

### Viral Infection

Like bacteria, many viruses causing infectious disease employ glycans on cell surface glycoproteins and glycolipids to access cells. These viruses often recognize unique glycan signatures on the glycocalyx, which frequently leads to specific tissue and species tropisms (25). An example is human adenoviruses (HAdV), one of the most common causes of ocular infection. Dozens of HAdVs, classified into seven species, A to G, have been identified on mucosal surfaces, but only a limited number of them cause epidemic keratoconjunctivitis in the eye (26). It appears that some of these viruses, primarily those belonging to species D, use sialic acid-containing glycans to establish infections at the ocular surface (27). One of them, the cornea-tropic HAdV-D37, specifically binds a branched oligosaccharide present in glycoproteins containing the GD1a glycan motif and featuring two terminal sialic acids (28). Coxsackievirus A24 variant (CVA24v) is another highly contagious infective agent that causes acute hemorrhagic conjunctivitis. Binding and infection experiments using corneal cells indicate that the cell surface receptor used by CVA24v is composed of a sialylated disaccharide (Neu5Acα2,3Gal) on O-glycosylated proteins (29). Consequently, derivatives based on sialic acid have been developed and shown to be effective in preventing HAdV-D37 and CVA24v binding and infection of human corneal epithelial cells (30, 31).

Efforts have been made to establish how these viruses bypass the transmembrane mucin-rich glycocalyx of the ocular surface epithelium to trigger infection and inflammation. The data suggest that, for successful infection, HAdVs need to degrade components of the mucin barrier. This is exemplified by HAdV-D37, which in contrast to HAdV-D19p, a virus that does not cause epidemic keratoconjunctivitis, releases the MUC16 ectodomain from corneal and conjunctival epithelial cells, causing a decrease in glycocalyx barrier function (32). On other occasions, transmembrane mucins contribute to masking viral entry mediators on the epithelial glycocalyx. Affinity assays have shown that herpes simplex virus type 1 (HSV-1), but not HSV-2, binds galectin-3, a component of the human corneal epithelial glycocalyx. Exposure of epithelial cell cultures to transmembrane mucin isolates decreases HSV-1 infectivity, suggesting that the strong association between transmembrane mucins and galectin-3 in the glycocalyx functions to mask the lectin and to provide protection against viral infection (33).

Many pathogens use glycosaminoglycans on the glycocalyx of host cells to initiate infection, and viruses are no exception. Heparan sulfate serves as a main HSV-1 entry receptor in the cornea and facilitates the development of herpetic keratitis (34, 35). Important to infection is the release of the viral progeny from the infected cells so that infection can disseminate into new target cells. Opposing this process are heparan sulfate chains on parent cells, which trap the exiting viral progenies and inhibit their release. Heparanase, a heparan sulfate-degrading enzyme, plays an essential role in facilitating viral egress. Following herpes infection of human corneal epithelial cells, heparanase promotes a continuous loss of heparan sulfate from the cell surface, leading to virus exit and subsequent tissue damage (36). This mechanism involves the upregulation of corneal epithelial heparanase by herpesvirus to promote enzymatic activity at the cell surface, as well as translocation of the enzyme to the nucleus, where it regulates downstream signaling pathways (37). The interaction of HAdV-D37 with sulfated glycosaminoglycans has also been recently reported. In these experiments, removal of heparan sulfate by heparinase III reduced HAdV-D37 binding to corneal epithelial cells but, at the same time, enhanced viral infection, leading the authors to hypothesize that glycosaminoglycans act as decoy receptors (38). Consequently, efforts have been made in the drug development field to use glycosaminoglycan mimetics as artificial decoy receptors that can inhibit HAdV-D37 cell attachment and infection (39).

### Fungal and Parasitic Infections

Fungal infections of the cornea can have devastating consequences if not treated promptly. They are frequently caused by species of *Fusarium*, *Aspergillus*, *Curvularia*, and *Candida*, with trauma being the most important predisposing cause (40). The first step for a successful infection is the presence of cell wall components and parasitic adhesins that mediate adhesion to host cells and components of the extracellular matrix. A number of interactions with host cells are known to lead to pathogen internalization, but the studies describing this invasion process remain scarce (41). Studies in corneal epithelium have revealed that *Cephalosporium curvulum* and *Aspergillus oryzae* use fucose-specific lectins to gain access to the host cell surface and, subsequently, promote infection and disease (42). It is worth noting here that a significant number of terminal and core fucose structures are present in the differentiated corneal epithelial glycocalyx, which could facilitate this type of interaction (43). In addition to immune cells, the corneal epithelium expresses lectins that also interact with polysaccharides on the fungal cell wall. An example is the C-type lectin dectin-1, which recognizes β-glucan. Binding of dectin-1 to *Candida albicans* initiates protective responses in the epithelium that include the regulation of the innate immune response through the dectin-1/NF-κB signaling pathway (44).

*Acanthamoeba* keratitis is a rare but serious parasitic infection of the cornea that can cause permanent vision loss. As previously stated for other microbes, adhesion to the surface of the host is one of the first steps in the pathogenesis of infection. Adhesion of *Acanthamoeba* is followed by lysis of corneal epithelial cells, degradation of extracellular matrix and penetration into the deeper layers of the cornea (45). A major virulence factor of this parasite is a mannose-binding protein that recognizes mannose-containing glycoproteins on the surface of the cornea and plays a role in promoting cytopathic effects (46). The presence of antibodies against the mannose-binding protein in tear fluid is thought to provide protection by inhibiting the adhesion of the parasite. Importantly, oral immunization with recombinant mannose-binding protein in an animal model has been shown to increase the levels of antibodies against this protein in tears and provide protection against *Acanthamoeba* keratitis (47).

## Controversies

The role of the epithelial glycocalyx in resisting or facilitating ocular infection is not free of controversy. As mentioned previously, the presence of highly glycosylated transmembrane mucins is thought to provide steric hindrance and limit *P. aeruginosa* adhesion to the host. On the other hand, using an azido GalNAc analog to label mucin-type O-linked glycoproteins, Jolly et al. observed that *P. aeruginosa* preferentially associated with GalNAc labeled-regions in the mouse cornea, leading the authors to conclude that surface glycosylation was not sufficient to prevent bacterial binding (48). It should be noted, however, that the metabolism of these azido sugars could pose significant drawbacks, including low specificity and the perturbation of the natural glycosylation process (49, 50). If this is the case, one could draw the opposite conclusion, i.e., that naturally occurring surface glycosylation is sufficient to maintain barrier function since perturbation of glycans physiologically present on the corneal surface, by using chemically modified monosaccharides, leads to bacterial binding.

MUC1 is one of the most extensively studied transmembrane mucins since it is known to regulate both pathogen invasion and the inflammatory response to infection. Assessment of the ocular surface of mice deficient in Muc1 revealed a marked propensity toward the development of irritation and inflammation (51). These mice were also more susceptible to bacterial infections based on the prevalence of *Staphylococcus*, *Streptococcus* and *Corynebacterium* species in infected eyes. Conversely, a parallel study using Muc1 null mice of a different genetic background found no particular ocular surface phenotype (52). Mouse eyes in the latter study had a normal appearance with no signs of ocular surface infection. Indeed, the authors could not find definitive evidence indicating that Muc1 null mice were more susceptible to *P. aeruginosa* adherence to the cornea. The differences in these studies were attributed to housing conditions of the animals, mouse strain variation, strain variation of pathogens or other environmental or epigenetic differences.

The roles of the different transmembrane mucins in protecting the human ocular surface epithelium have been the subject of intense study. While it was assumed for many years that they play a redundant function in protecting the underlying tissue against environmental insult, new evidence appears to challenge this concept. Experiments *in vitro* have evidenced that MUC16 in human corneal epithelial cells prevents bacterial adherence and invasion, while the smaller MUC1 does not (53). Surprisingly, abrogation of MUC1 in these experiments enhanced the barrier with respect to bacterial adherence and invasion, leading the authors to hypothesize that MUC16 alone produces a more uniform, glycan-rich barrier on the epithelial glycocalyx.

## Concluding Remarks

**Figure 1:**
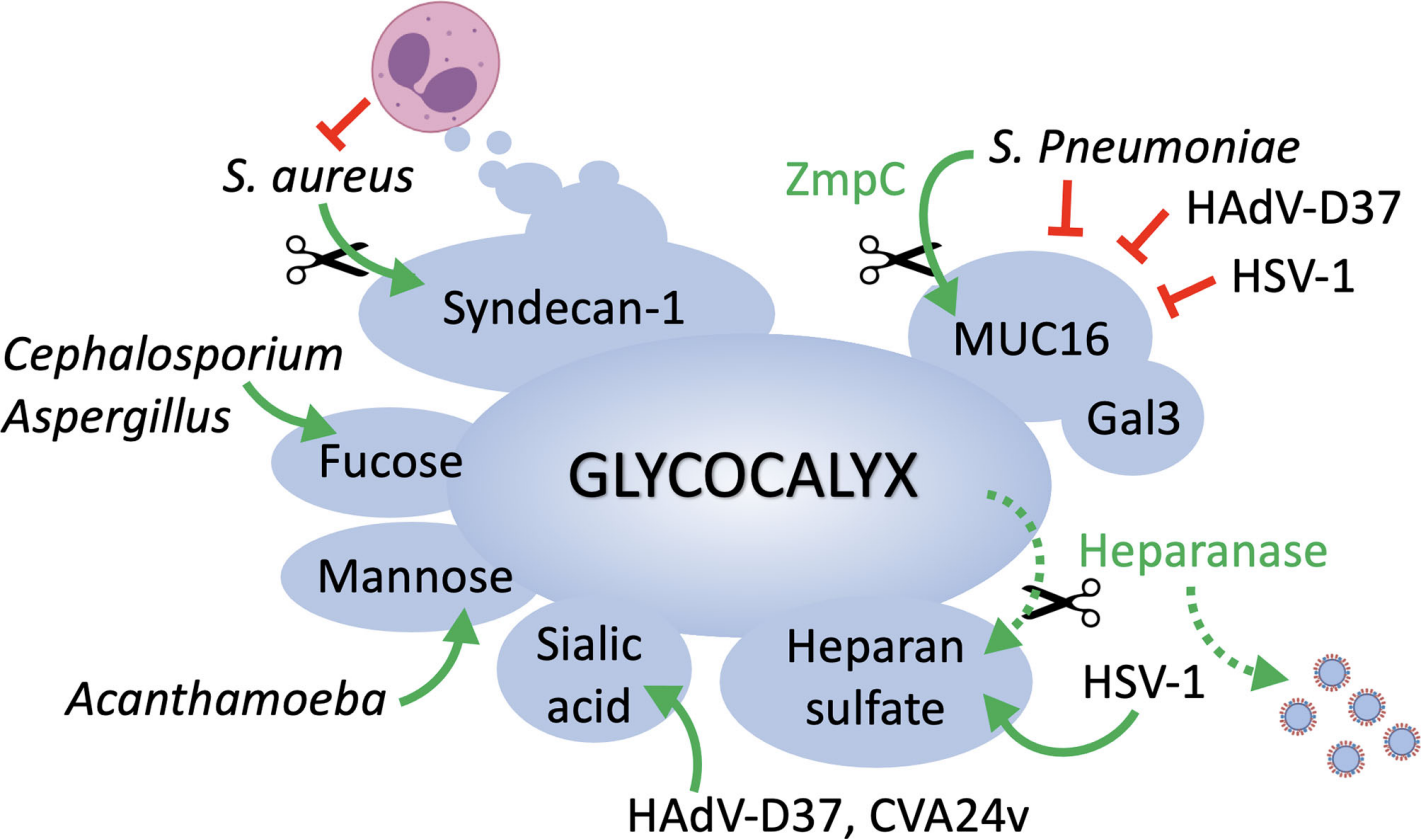
The epithelial cell glycocalyx in ocular surface infection. MUC16 represents one of the first barriers to the passage of bacteria and viruses at the ocular surface, and its function is reinforced by galectin-3. Pathogens must rely on the enzymatic removal of mucins (e.g., ZmpC) to access epithelial cells and cause disease. Pathogens such as *S. aureus* circumvent immune cell destruction by inducing shedding of syndecan-1 from the cell surface. HSV-1 uses heparan sulfate as a main entry receptor but also promotes heparanase biosynthesis in host cells to facilitate the release of viral progenies. Cell surface glycans containing sialic acid, mannose or fucose serve as receptors for viral, fungal and parasitic organisms.

Multiple studies have evidenced over time the extent of glycosylation at the ocular surface epithelia and its relevance to infection (**Figure 1**). Both corneal and conjunctival epithelial cells contain a complex glycocalyx rich in transmembrane mucins, proteoglycans and glycosphingolipids. Adhesion of pathogens to glycans present in some of these molecules is one of the first steps leading to the successful colonization of the eye. At the same time, the ocular surface exhibits protective glycoconjugates that actively prevent pathogen invasion. One of them is transmembrane mucins that extend high above other molecules present on the glycocalyx, thereby providing a physical barrier and masking receptors by steric hindrance. In addition, the ocular surface fights infection by exposing host molecules that act as decoy receptors, impeding pathogen adhesion and the initiation of signaling cascades.

Contact lens wear, trauma, and ocular surface disease constitute common risk factors that predispose patients to infections. Interestingly, most of these factors have been associated with alterations in glycosylation. Carbohydrate moieties on cell surface glycoproteins change in response to corneal wounding and contact lens wear, and inflammatory stimuli of the ocular surface epithelia induce alterations in mucin-type O-glycosylation and N-glycan processing (43, 54–56). It would be noteworthy to determine if these changes in glycosylation contribute to the higher risk of ocular infection. Other exciting areas that remain understudied in the eye include the influence of genetic factors (e.g., secretor status) on disease susceptibility and the role of ocular surface glycans in controlling microbial stability or virulence (57, 58). This information may be useful for designing effective prophylactic and therapeutic agents targeting the microbe–host interface.

## Author Contributions

All authors listed have made a substantial, direct, and intellectual contribution to the work and approved it for publication.

## Funding

We acknowledge the funding support from the National Institutes of Health, NEI Grants R01EY026147 and R01EY030928.

## Conflict of Interest

The authors declare that the research was conducted in the absence of any commercial or financial relationships that could be construed as a potential conflict of interest.

## Publisher’s Note

All claims expressed in this article are solely those of the authors and do not necessarily represent those of their affiliated organizations, or those of the publisher, the editors and the reviewers. Any product that may be evaluated in this article, or claim that may be made by its manufacturer, is not guaranteed or endorsed by the publisher.
